# Qishen Yiqi dripping pills for ischemic heart failure: A protocol for a prospective cohort study

**DOI:** 10.1097/MD.0000000000013085

**Published:** 2018-11-09

**Authors:** Zhiqiang Zhao, Xianliang Wang, Yazhu Hou, Shuai Wang, Jingbo Zhai, Cong Wang, Jingyuan Mao, Boli Zhang

**Affiliations:** a Cardiovascular Department of First Teaching Hospital of Tianjin University of Traditional Chinese Medicine; b Tianjin University of Traditional Chinese Medicine, Tianjin, China.

**Keywords:** ischemic heart failure, patients’ preference, Qishen Yiqi dripping pills, traditional Chinese medicine

## Abstract

**Background:**

The prognosis of ischemic heart failure (IHF) is worse than non-IHM. Improving the management of IHF remains an urgent demand. In recent years, Qishen Yiqi dripping pills (QSYQ), a type of Chinese herbal medicine (CHM), has been popular for IHF combined with standard western medicine. However, relevant scientific evidence from the real clinical practice still is insufficient. The prospective cohort study aims to assess the effectiveness and safety of QSYQ plus standard western medicine for IHF in the real clinical practice.

**Methods:**

It is a multicenter, prospective, observational cohort study. A total of 1200 patients with IHF recruited from 84 hospitals in China will be assigned to exposure group (patients with QSYQ treatment) or non-exposed group (patients without QSYQ treatment) mainly according to patients’ preference in real clinical situation. The primary outcomes include New York Heart Association (NYHA) cardiac functional classification and Minnesota Living with Heart Failure Questionnaire (MLHFQ). The secondary outcomes include composite outcomes (all-cause mortality, frequency of re-admission or emergency due to cardiovascular events), left ventricular ejection fraction and cardiothoracic ratio, symptoms and signs obtained by the 4 Tradiational Chinese Medicine (TCM) diagnostic methods. Assessments will be performed at baseline, 1st and 3rd month after enrollment.

**Discussion:**

It will provide new evidence on QSYQ for IHF in real clinical practice.

**Study registration:**

This study has been registered on the Chinese Clinical Trial Registry (No: ChiCTR-ONRC-14004407).

## 1 Background

Heart failure (HF) has become a major public health problem associated with high prevalence, poor prognosis and huge economic burden despite recent improvements of its treatment.^[1]^ Ischemic heart failure (IHF) is the most common type of HF. The prognosis of patients with IHF is worse than those with other types of HF.^[2–4]^ Improving the management of IHF remains an urgent demand.

Chinese herbal medicine (CHM) is popular for treating patients with IHF combined with routine western medicine in China.^[5]^ Qishen Yiqi dripping pills (QSYQ) consisting of active ingredients from Huangqi, Danshen, Sanqi, and Jiangxiang and it is a type of CHMs produced by Tasly Pharmaceutical Co. Ltd and approved for ischemic heart disease by China State Food and Drug Administration in 2003. Experimental research suggests QSYQ could be beneficial for IHF through multiple mechanisms such as inhibiting platelet aggregation, enhancing cardiac function, reducing serum brain natriuretic peptide (BNP). preventing cardiac ventricular remodeling, and so on.^[6–12]^ A 2014 systematic review suggests QSYQ could improve the cardiac function in patients with IHF without severe side-effects.^[13]^ However, current evidence from randomized clinical trials (RCTs) still remains inconclusive due to poor methodological quality such as small sample size, single center, and so on,^[13]^ and the external authenticity is limited in view of patients’ preference for treatment in real clinical practice.^[14]^ In this prospective cohort study, we aim to assess the effectiveness and safety of the combination of QSYQ and routine western medication for IHF considering patients’ preference in the real world.

## 2 Methods

### 2.1 Study design

**Figure 1:**
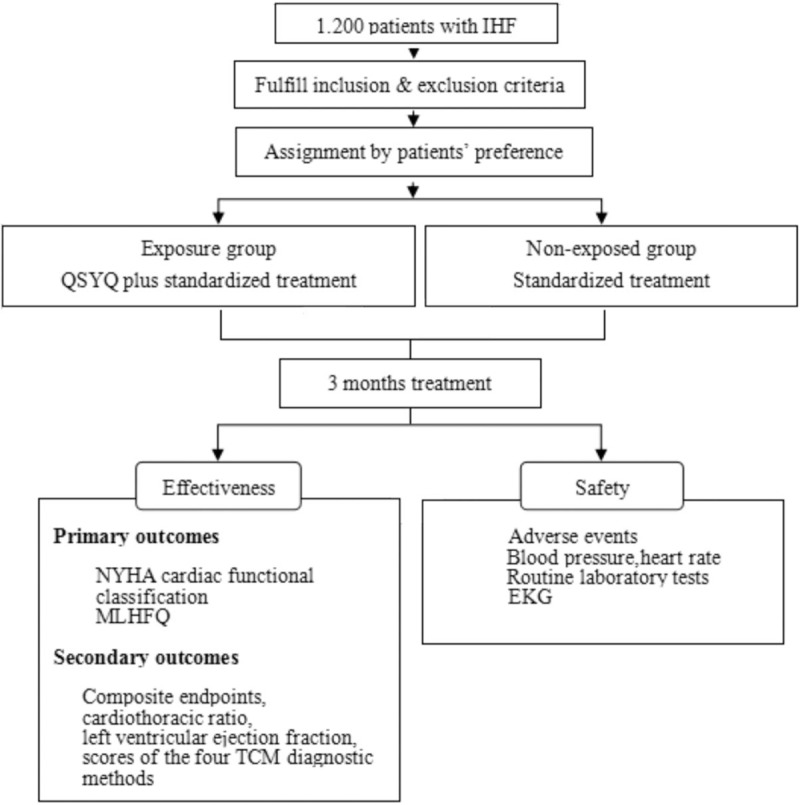
Flow diagram of study.

It is a multicenter, prospective, observational cohort study. A total of 1200 outpatients with IHF from 84 hospitals will in different regions of China are divided into 2 cohorts: exposure group (patients with QSYQ treatment) and non-exposed group (patients without QSYQ treatment). The choice for groups will be mainly made according to patients’ preference. Assessments will be performed at baseline, 1 and 3 months after enrollment. Outcome assessors and statisticians responsible for the final analysis will be blinded. The details of the flow diagram are shown in Figure 1.

### 2.2 Ethics

The protocol has been approved by the Ethics Committee of First Teaching Hospital of Tianjin University of traditional Chinese medicine (NO: TYLL2013 [K] 008). It will be conducted in accordance with the Declaration of Helsinki. Written informed consent will be obtained from all participants or their legally authorized representatives when they agree to participate in the study before enrollment.

### 2.3 Participants

#### 2.3.1 Diagnostic criteria

The patients’ diagnostic criteria are based on European Society of Cardiology guidelines for the diagnosis and treatment of acute and chronic HF 2012 and 2007 Chinese Society of Cardiology guidelines for the diagnosis and treatment for chronic stable angina.^[15,16]^ The definition of IHF refers to HF with the history of coronary heart disease. Patients matching 1 of the following 3 conditions will be diagnosed as coronary heart disease:

(1) with prior or current symptoms, such as dyspnea, fatigue and fluid retention (edema), so on;
(2) history of old myocardial infarction with or without percutaneous coronary intervention or/and coronary artery bypass grafting;
(3) coronary angiography or coronary Computed Tomography Angiography (CTA) shows stenosis more than 50% in at least 1 of the main coronary arteries with or without revascularization.

#### 2.3.2 Inclusion criteria

(1) Diagnosed with coronary heart disease;
(2) Left ventricular ejection fractions (LVEF) ≤50%;
(3) New York Heart Association (NYHA) classification II∼IV;
(4) Standardized western medicine therapy for IHF currently in a stable condition;
(5) Signed informed consent.

#### 2.3.3 Exclusion criteria

(1) Acute HF;
(2) Acute coronary syndrome;
(3) Preparation for revascularization therapy or heart transplantation;
(4) Already received or preparing to receive cardiac resynchronization therapy (CRT)/implantation of cardiac pacemaker;
(5) Special treatments should be used for patients with comorbidities such as severe liver or renal failure and malignant tumor which could influence the clinical treatment of IHF;
(6) Pregnant or breastfeeding women, or women at childbearing age without reliable methods of contraception;
(7) Participated in other studies within 2 months;
(8) Suspected or definite allergy to intervention drugs.

### 2.4 Sample size

According to a previous study,^[17]^ we assume that the rate of cardiac function improvement is 22% of patients in QSYQ group and 15% in control group. The sample size is same in 2 groups. A total sample size of 1200 patients should be recruited with a power of 0.8, a 2-sided alpha of 0.05 and a dropout rate of 20% by PASS 2011 software.

### 2.5 Intervention

The patients will be divided into 2 cohorts: exposure group and non-exposed group according to whether are taking QSYQ in real clinical practice. Patients receive treatments from researchers of QSYQ or not as they would in the real world, and if they have no preference, the decision for group will be made by researchers according to their professional experience. Patients in the non-exposed group will receive standardized western medication according to patient's clinical conditions, which includes antiplatelet agents, statins, diuretics, angiotensin-converting enzyme inhibitor or angiotensin-receptor blocker, β-blockers, aldosterone receptor antagonist, digoxin, vasodilating agents, and so forth according to HF and chronic stable angina guidelines.^[15,16]^ Patients in the exposure group will be given 1 packet (0.52 g) of QSYQ thrice daily 3 months besides standardized western medication. In both groups, any other Tradiational Chinese Medicine (TCM) drug will be prohibited. Western medicine will be administrated for the comorbidities (such as hypertension or diabetes) and complications according to the relevant guidelines. All drugs will be recorded in case report form.

### 2.6 Outcome measures

#### 2.6.1 Primary outcomes

The primary outcomes include NYHA cardiac functional classification and Minnesota Living with Heart Failure Questionnaire (MLHFQ). They will be assessed at screening (V0 phase), 1 Month ± 7 days (V1 phase), and 3 Month ± 7 days (V2 phase).

#### 2.6.2 Secondary outcomes

(1) Composite outcome, including all-cause mortality, frequency of re-admission or emergency due to cardiovascular events (exacerbated HF, acute coronary syndrome, malignant arrhythmias, cardiac shock, coronary revascularization, stroke, pulmonary embolism, peripheral vascular events, etc.) recorded in V1 and V2.
(2) LVEF estimated by Simpson's method and cardiothoracic ratio will be assessed at V0 and V2.
(3) Symptoms and signs from the 4 TCM diagnostic methods will be measured at V0, V1, and V2.

The entire study period for each patient will be 3 months. All the visits and items are presented in Table 1.

**Table 1:**
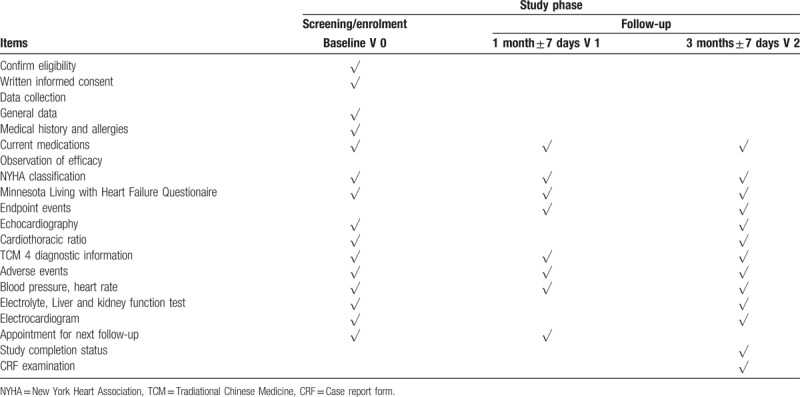
Time schedule of participant enrollment and follow-up.

### 2.7 Safety outcomes

Vital signs, some laboratory tests and adverse events are considered as safety outcomes. Vital signs, which including blood pressure and heart rate, will be measured at V0 and V2. Routine laboratory tests (routine urinalysis, hepatic and renal function and serum electrolyte profile) and electrocardiograms will be measured at V0 and V2. The adverse events will be documented at each visit. The prognosis of adverse events would be observed until the adverse reactions disappear or relieve.

### 2.8 Data collection

All data will be documented in the case report form. At the end of the study, the researchers, data managers, and statisticians will review and confirm the database for the final statistical analysis.

### 2.9 Statistical analysis

Statistical analysis will be performed by the Clinical Trials Center of First Teaching Hospital of Tianjin University of TCM based on intention-to-treat principle. The continuous variables will be expressed as the mean with standard deviation and tested by *t* tests or Wilcoxon rank sum test. Categorical variables will be expressed as percentages and tested by chi-squared test or Fisher exact test. Survival analysis will be conducted by the log-rank test and Cox regression model. Statistical analysis on the missing data will be adjusted by an estimating equation or statistical model in the final analysis. The statistical significance is defined as *P* <.05. Data analysis will be conducted by Statistical Analysis System (SAS) software version 9.3 (SAS Institute Inc., Cary, NC).

## 3 Discussion

In China, QSYQ plus western medicine is widely used for patients with IHF, especially for those who are dissatisfied with the efficacy or want to relieve the side effects from western medication. Current evidences on QSYQ for IHF mainly come from RCTs with poor methodological quality.^[13]^ The external authenticity is limited when patients have preferences for treatments in RCT design.^[14]^ In this context, we design a multicenter, prospective, observational cohort study considering the patients’ preference in the real clinical practice. It will provide new evidence on QSYQ for IHF in real clinical practice.

## Acknowledgments

We gratefully acknowledge the contributions of the following hospitals: Hospital of Wangjiang County of Anhui Province, The people's Hospital of Linquan County, The people's Hospital of Jinzhai County, Wuhu Hospital of Traditional Chinese Medicine, The people‘s Hospital of She County, Dongshan County Hospital of Traditional Chinese Medicine, Gutian County Hospital, The Hospital of Anguo City, The people's Hospital of Nanpi County, The People's Hospital of Pingshan County, The first Hospital of Shijiazhuang City, The people's Hospital of Suning County, Tangshan City Hospital of Traditional Chinese Medicin, The People's Hospital of Zunhua City, The people's Hospital of Wei County, The Hospital of Yutian County, The Central Hospital of Dengzhou City, Minquan County Hospital of Traditional Chinese Medicine, Xinye County Hospital of Traditional Chinese Medicine, The People's Hospital of Qinyang City, Xinmi City Hospital of Traditional Chinese Medicine, The People's Hospital of Shangcai County, The people's Hospital of Xiangcheng County, The Second Affiliated Hospital of Zhengzhou University, The people's Hospital of Dongning County, The people's Hospital of Hailin, The people's Hospital of Suiling County, The people's Hospital of Daxinganling, Qiqihar Hospital of Traditional Chinese Medicine, The third hospital of Heilongjiang Province, Infectious disease control and prevention Hospital of Heilongjiang Province, The people's Hospital of Zhijiang City, Yiling Hospital of Yichang City, The central Hospital of Suizhou City, The first people's Hospital of Tianmen City, Liyuan Hospital Affiliated to Tongji Medical College of Huazhong University of Science and Technology, Puren Hospital of Wuhan City, The People's Hospital of Yunxi County, The People's Hospital of Yun County, Hengdong County Hospital of Traditional Chinese Medicine, The first people's Hospital of Pingjiang County, The people's Hospital of Wangcheng District of Changsha City, The people's Hospital of Xiangxiang City, The central Hospital of Baishan City, Dehui City Hospital of Traditional Chinese Medicine, Hunchun City Hospital, The people's Hospital of Huinan County, The people's Hospital of Jiutai City, The people's Hospital of Jiangyuan District of Baishan City, The Centeal Hospital of Meihekou City, Qianguo County Hospital, The central Hospital of Tonghua City, The General Hospital of SINOHYDRO BUREAU I CO.LTD., Jintan City Hospital of Traditional Chinese Medicine, Liyang City Hospital of traditional Chinese Medicine, Lishui District Hospital of Traditional Chinese Medicine of Nanjing City, The second people's Hospital of Taizhou City, Funing County Hospital of Traditional Chinese Medicine, Rugao City Hospital of Traditional Chinese Medicine, The people's Hospital of Xiangshui County, The first people's Hospital of Zhangjiagang City, The people's Hospital of Gaoan City, Rehabilitation hospital of Jianping County, Huangzhong County Hospital of Traditional Chinese Medicine, The people's Hospital of Minhe County, The third people's Hospital of Xining City, The people's Hospital of Yi’nan County, The people's Hospital of Shouyang County, The Third Hospital of YANGQUAN COAL INDUSTRY(GROUP)CO.,LTD, The Tianjin Hospital of Ningqiang County, The people's Hospital of Fufeng County, The people‘s Hospital of Shenmu County, Shanghai Renhe Hospital of Baoshan District, The people's Hospital of Pi County, The first Hospital of Tianjin City, The 254 Hospital of People's Liberation Army, Nankai Traditional Chinese Medicine Hospital of Tianjin, Tianjin Hospital of ITCWM Nankai Hospital, The Third Hospital of Tianjin, Beichen Traditional Chinese Medicine Hospital of Tianjin, The people's Hospital of Haining City, The people's Hospital of Qingtian County, The people's Hospital of Lanxi City of Zhejiang Province, The first people's Hospital of Tongxiang City.

## Author contributions

ZQZ, XLW, JYM, and BLZ conceived the research question and designed the study. YZH and JBZ contributed to the design of the study and review of the manuscript as clinical experts. ZQZ and XLW drafted the manuscript. SW and CW are responsible for data curation. JYM critically reviewed the overall manuscript and takes full responsibility for the study. All authors have read and approved the final manuscript.

**Conceptualization:** Xianliang Wang, Jingyuan Mao, Boli Zhang.

**Data curation:** Shuai Wang, Cong Wang.

**Funding acquisition:** Jingyuan Mao.

**Methodology:** Zhiqiang Zhao, Jingyuan Mao, Boli Zhang.

**Writing – original draft:** Zhiqiang Zhao, Xianliang Wang.

**Writing – review & editing:** Yazhu Hou, Jingbo Zhai, Jingyuan Mao.
